# Comparison of Inductively Coupled Plasma Optical Emission Spectrometry with an Ion Selective Electrode to Determine Sodium and Potassium Levels in Human Milk

**DOI:** 10.3390/nu10091218

**Published:** 2018-09-03

**Authors:** Ching Tat Lai, Hazel Gardner, Donna Geddes

**Affiliations:** School of Molecular Sciences, University of Western Australia, 35 Stirling Highway, Crawley, 6009 WA, Australia; hazel.gardner@uwa.edu.au (H.G.); donna.geddes@uwa.edu.au (D.G.)

**Keywords:** human milk, potassium, sodium, ICP-OES, ion selective electrode

## Abstract

Sodium (Na), potassium (K), and the ratio Na:K in human milk (HM) may be useful biomarkers to indicate secretory activation or inflammation in the breast. Previously, these elements have been measured in a laboratory setting requiring expensive equipment and relatively large amounts of HM. The aim of this study was to compare measurements of Na and K in HM using inductively coupled plasma optical emission spectrometry (ICP-OES) with small portable ion selective electrode probes for Na and K. Sixty-five lactating women donated 5 mL samples of HM. Samples were analyzed with two ion selective probes (Na and K) and also ICP-OES. The data were analyzed using paired *t*-test and Bland–Altman plots. Na concentrations were not significantly different when measured with ion selective electrode (6.18 ± 2.47mM; range: 3.59–19.8) and ICP-OES (5.91 ± 3.37 mM; range: 2.59–21.5) (*p* = 0.20). K concentrations measured using the ion selective electrode (11.7 ± 2.21 mM: range: 7.69–18.1) and ICP-OES (11.1 ± 1.55 mM: range: 7.91–15.2) were significantly different (*p* = 0.01). However, the mean differences of 0.65 mM would not be clinically relevant when testing at point of care. Compared to ICP-OES, ion selective electrode is sufficiently accurate to detect changes in concentrations of Na and K in HM associated with secretory activation and inflammation in the mammary gland.

## 1. Introduction

Human milk (HM) sodium (Na) and potassium (K) concentrations change dramatically during the first week postpartum at the onset of secretory activation. Sodium, in particular, follows a rapid downward trajectory after birth as a result of tight junction closure, which is essential for secretory activation of the onset of copious milk production [1]. It has been reported that human milk sodium drops from 60 mM to 10 mM between days 1 and 5 postpartum, this precipitous drop reflects tight junction closure [2]. Delayed onset of secretory activation is established as a risk factor for poor lactation outcomes [3] with high sodium concentrations at day 7 considered a risk factor for the cessation of breastfeeding [4].

The Na:K ratio is another marker that has been historically used to define secretory activation. The Na:K ratio is greater than 2.0 after birth and then declines as sodium concentrations decline with closure of the tight junctions. The Na:K ratio has been used to biochemically define lactation stages as follows: ≥2 colostral milk, <2 transitional milk, and <0.6 mature milk [1,5]. This is mirrored by changes in the human milk transcriptome during this period and is therefore considered more accurate than using time postpartum to define lactation stage [5]. Changes in the Na:K ratio form a continuum over the first week postpartum, with a high Na:K ratio on day 7 (>0.8) [4] indicative of suboptimal milk supply or feeding problems and consequently a higher risk of breastfeeding cessation. An elevated ratio on day 7 is associated with 3.3 times greater odds of stopping breastfeeding in mothers reporting concerns about milk supply [4].

A high Na:K ratio is also indicative of the increased mammary epithelial permeability associated with breast inflammation, breast engorgement or mastitis [4,6,7,8]. The Na:K ratio is viewed as superior to sodium alone as it negates the variation found in proportions of aqueous and fat layers of the milk which occur when different sampling methods are used. Interestingly, the Na:K ratio has been found to be positively associated with the inflammatory chemokine interleukin-8 concentrations suggesting that this ratio is also a marker for inflammation of the mammary epithelium [7].

Immediate measurement of HM sodium and potassium concentrations would be advantageous in situations of suspected delayed secretory activation and mammary infection allowing rapid intervention. Currently these components are measured with instruments such as flame photometry [4], atomic absorption spectroscopy [7], ion chromatography, inductively coupled plasma optical emission spectroscopy (ICP-OES), and inductively coupled plasma mass spectroscopy (ICP-MS). All of these techniques, which require hazardous chemicals, laboratory facilities, and highly skilled operators, are not immediate and are also costly. Alternatively, the ion selective electrode (ISE) is easy to use, portable, and provides immediate results and therefore has potential as a point of care device for screening of high-risk lactating women. Ion specific electrodes have been used previously, but have not been compared directly to other methods to determine their accuracy [9].

The objective of this study was to compare the accuracy of the sodium and potassium ion selective electrodes against inductively coupled plasma optical emission spectrometry (ICP-OES).

## 2. Materials and Methods

### 2.1. Biochemical Analysis

Sixty-five preterm/term mothers provided written informed consent to participate in the study, which was approved by the Human Ethics Committee at the University of Western Australia (RA/4/1/2369). Five mothers who participated in this study had a premature delivery (<37 weeks gestation). Each participant completed a 24 h milk profile using the test weighing method [10]. Mothers were issued with a set of baby weigh scales (Medela AG, Baar, Switzerland). Samples were collected in 5 mL polypropylene tubes (P5016SL, Techno Plas Pty Ltd, St Marys, SA, Australia) before and after each feed or breast expression. Milk samples collected from the 24 h milk production were then pooled and approximately 5 mL of the pooled samples were used in this study.

#### 2.1.1. Ion Selective Electrode Measurement of HM Na and K

Concentrations of sodium and potassium in the milk samples were determined by ion selective electrodes (Sodium: B-722; potassium: B-731; Horiba, Japan). Calibration of the electrodes was conducted according to the manufacturer’s recommendations. The calibration range for both Na and K electrodes were set between 150 to 2000 ppm according to the manufacturer’s instructions. For each assay, the whole milk samples were thawed at 37 °C for 1 h. Prior to the measurement, the milk samples were shaken with an Intelli-mixer (RM-2, ELMI Ltd, Riga, Latvia) using tube stand mode for 15 s at 50 rpm followed by 3 inversions. Three hundred microliters of the mixed milk was pipetted onto the electrode sensor. The sample was allowed to stabilize for 15 s before the reading was taken. After each measurement, the milk sample was removed from the sensor and returned to the storage tube. The sensor was then rinsed with double deionized water and wiped with KimWipes (Kimberly-Clark Worldwide, Irving, TX, USA) prior to the next measurement. All samples were analyzed together, in duplicate. The same procedures were applied to both electrodes. The results of Na and K obtained from the electrodes were averaged and were converted from ppm to mM using the molecular weight of Na and K (23 and 39 g/mol, respectively).

#### 2.1.2. Inductively Coupled Plasma Optical Emission Spectrometry (ICP-OES) Measurement of HM Na and K Reagents

Standard solutions of Na and K (High Purity Standard, USA, 1000 µg/mL) were in the range of 50–500 µg/mL. Yittrium and scandium were used as internal standards (1000 µg/mL) from Sigma Aldrich (Castle Hill, NSW, Australia). All standards were diluted with 18.2 MΩ water.

##### Sample Preparation and Measurement

Whole milk or standard solution (200 µL) was mixed with 300 µL of nitric acid (65%, Suprapur^®^, Merck, Kenilworth, NJ, USA) into disposable borosilicate glass tubes (10 × 75 mm, Kimble Chase, Rockwood, TN, USA). The tubes were placed into a dry heating block (DBH40D, Ratek, Boronia, Victoria, Australia) and a dry acid washed marble was placed on top of the tube. The tubes were incubated at 110 °C for 1 h to allow for the completion of acid digestion. The hot tubes were allowed to cool down in an ice bath before adding the internal standard (2.1 µg/mL) and topped up to 2 mL with 18.2 MΩ water. The concentrations of Na and K in the digested milk samples were determined in triplicate by ICP-OES (5100, Agilent technologies, Santa Clara, CA, USA). Na and K were detected and measured at 568.263 nm and 568.821 nm for Na; and 766.491 mM and 769.897 mM for K. The results of Na and K obtained from those specific wavelengths were averaged and were converted from µg/mL to mM using the molecular weight of Na and K (23 and 39 g/mol, respectively).

#### 2.1.3. Validation of Analytical Methods

Both methods were validated using a spike/recovery assay. In each set of recovery assay, there was 3 tubes: (a) known standard solution + milk; (b) water + milk; (c) known standard solution + water. The mixture in each tube was 1:1 (v:v). The concentrations of Na or K in the tubes were measured and the following formula was applied: ((a)−(b))/(c) × 100% to obtain the recovery (%) for the set. Five sets were measured for each method to ensure the recovery was between 95–105% with a coefficient of variation (CV) <10%. The recovery (%) for the electrode method was 95 ± 4.1 for Na and 99 ± 3.9% for K with a CV of 4.1% and 3.9%, respectively (*n* = 5), and for ICP-OES was 99.0 ± 9.9 for Na and 99 ± 9.3% for K with a CV of 9.9% and 9.4%, respectively (*n* = 5).

### 2.2. Statistical Analysis

Statistical analyses were carried out using R 3.4.4 [11] and R Studio 1.1.419 [12] with package Lattice [13] for Bland–Altman plots. A paired sample *t*-test was used the compare the Na or K concentrations between the two methods. The limits of agreement and the precision of the estimated limits of agreement between the two measuring methods were calculated [14]. Bland–Altman plots were created to illustrate the limits of agreement. Pearson correlation was used to determine the correlation between concentrations of Na or K measured by the two methods. Boxplots were used to illustrate the medians, quartiles, and the 5th and 95th percentiles. The results were expressed as mean ± standard deviation (SD) unless stated otherwise. Differences were considered significant if *p <* 0.05.

## 3. Results

Sixty-five participants were recruited to include a range of lactation stages and milk productions as shown in Table 1.

**Figure 1:**
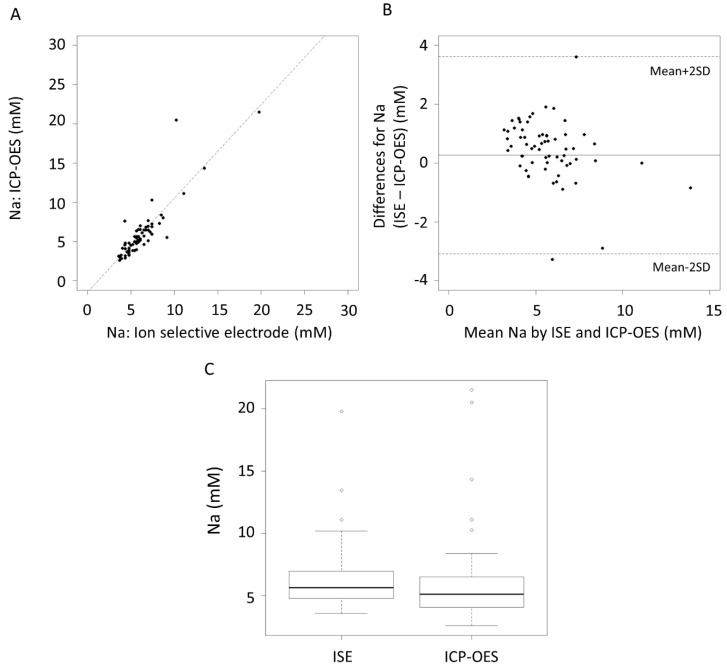
(**A**) Correlation of Na concentrations as measured by the ion selective electrode and inductively coupled plasma optical emission spectrometry (ICP-OES), *r*^2^ = 0.76, *p* < 0.001. (**B**) Bland–Altman plot showing the mean differences and limits of agreement. (**C**) Boxplot of Na concentrations, ion selective electrode (ISE).

The correlation coefficient (*r*^2^) of the Na measurement between the ISE and ICP-OES was 0.76, *p* < 0.001 (Figure 1A). The mean difference in the Na measurement between ISE and ICP-OES was within ±2 standard derivations (SD) (Figure 1B). There was no significant difference between the measurement of Na between the ISE and ICP-OES (*p* = 0.20, Figure 1C).

**Figure 2:**
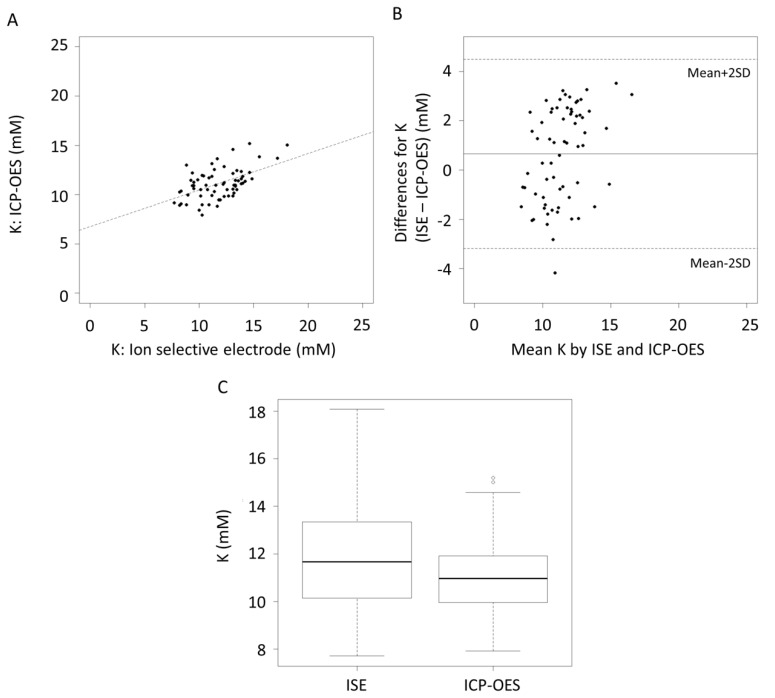
(**A**) Correlation of K concentrations as measured by the ion selective electrode and ICP-OES, *r*^2^ = 0.26, *p* < 0.001. (**B**) Bland-Altman plot showing the mean differences and limits of agreement. (**C**) Boxplot of K concentrations.

The correlation coefficient (*r*^2^) of the K measurement between ISE and ICP-OES was 0.26, *p* < 0.001 (Figure 2A). The mean difference in the K measurement between ISE and ICP-OES was within ±2 standard deviations (SD) (Figure 2B). Measurement of K by the K ISE was significantly higher than ICP-OES (*p* = 0.01, Figure 2C).

Twelve scenarios were considered (high to low measures for both Na and K) to evaluate the impact of the change in the measurement of K on the Na:K ratio as shown in Table 2. In all 12 scenarios a movement equivalent to the mean difference in K is not enough to move the Na:K ratio over either the 0.8 or 2 thresholds.

## 4. Discussion

Inductively coupled plasma optical emission spectrometry is regarded as one of the “gold standard” techniques for trace element analysis. It utilizes specific wavelengths to detect and measure Na (568.263 nm and 568.821 nm) and K (766.491 nm and 769.897 nm) in the acid digested fluid, in this case HM, during the process. We found no statistically significant differences between Na concentrations as measured by the Na specific electrode and the ICP-OES in 65 whole HM samples (Figure 1C, Table 3).

For K, measurements were significantly higher on average with the ion specific electrode (ISE) compared to the ICP-OES measurements (Figure 2C, Table 3). Whilst the ISE and ICP-OES were significantly correlated (Figure 2A) and the values fell within 2 SD of the mean (Figure 2B) the confidence intervals of the mean difference also suggest a significant difference between the two methods. The design of the ISE for Na and K is based on a polymeric membrane such that the size of the molecular cavity on the membrane matches the size of the targeted ion for the measurement [15]. The only difference between Na and K ion selective electrodes is the type of polymeric membrane used. The Na ion selective electrode membrane is sodium ionophore II, while valinomycin is the polymeric membrane for the K ion selective electrode. The mean difference of 0.65 mM in the measurement of K between the two methods may be related the effect of the milk matrix [16,17]. Components in milk, such as proteins and other ions, could interfere with the interaction of K and the membrane of the ISE by partially blocking some of the molecular pores in the membrane. The matrix effect of milk may be more prominent on the ISE of K as the molecular cavity of the membrane of the K ISE is greater than that of the Na ISE.

Nevertheless, the measurement of Na and K with both methods was comparable and the mean differences of Na and K with both methods were within the limits of agreement (Table 4). Furthermore, within the first year of lactation, the mean concentrations of Na and K in milk ranged between 11 to 60 mM and 4 to 18.2 mM, respectively [18]. We calculated Na:K ratios using all permeations of high, medium, and low levels of Na and K to determine if the ratio would shift dramatically to alter diagnosis particularly in the case of identifying secretory activation. Indeed, we found the ratio not to be altered enough to change a clinical diagnosis. (Table 2).

The accuracy of the ISE was comparable to ICP-OES with a significant correlation (Figure 1A and Figure 2A) and good limits of agreement (Figure 1B and Figure 2B). The ISE could therefore be used to measure the high sodium concentrations found in antepartum secretions, colostrum, and the milk from mastitic breast or milk during the involution phase [6,19]. Under all these conditions the integrity of the tight junctions is compromised resulting in widening of the paracellular pathway between the mammary epithelial cells allowing transfer of components between the circulation and milk [19].

Human milk sodium concentrations decrease rapidly in the first three days post-partum as secretory activation occurs in response to the withdrawal of progesterone. However, milk sodium levels have a nonlinear relationship with milk volume, suggesting changes are not due to dilution as milk volume increases [1]. Delayed secretory activation is a risk factor for reduced breastfeeding success [1]. HM sodium drops from 60 mM to 10 mM between days 1 and 5 postpartum [2]. As such, the ion selective probe is accurate enough to monitor HM sodium levels postpartum to confirm secretory activation particularly in high risk mothers such as primiparous mothers, those with maternal obesity, preterm birth, caesarean deliveries, and those who have had long and complicated deliveries [20,21,22,23,24].

Low milk supply is also a frequent concern for mothers, particularly primiparous mothers [3,25]. This concern may be either perceived or actual milk supply insufficiency. It is known that milk production at week 2 is predictive of milk production at week 6; therefore, the first 14 days are critical to the establishment of a good milk supply. Na:K ratios during the first week postpartum have been shown to be useful biochemical indicators of suboptimal milk production, and as a result, are predictors of shorter breastfeeding duration [4,9,26]. High Na:K ratios are indicative of incomplete tight junction closure. This may impact the volume of milk produced and thus the transition to full lactation [9]. Higher breastfeeding frequency is associated with lower sodium levels, and increased production. Dewey et al. [20] recommend that all mother and infant dyads be followed up at 72–96 h postpartum to ensure that secretory activation has occurred and a biochemical indicator such as ion selective probes that measure Na and K may provide rapid results to ensure early intervention to improve milk production.

The ISE may also be useful in immediate detection of mastitis particularly subclinical mastitis [27] via increases in Na:K (>1.0). Mastitis is a debilitating inflammatory breast disease, reported to result in cessation of breastfeeding in as many as 20% of cases; therefore, rapid detection would allow early treatment and resolution resulting in better breastfeeding outcomes. HM sodium levels decrease over time with mature milk to 4–5 mM, whereas HM Na levels of 12 mM and upwards are indicative of subclinical mastitis and mastitis. The Na:K ratio has been categorized as <0.6 normal, ≥0.6 and ≤1 slightly elevated, >1 very high [7]. Na:K above 1.0 is commonly used in the diagnosis of subclinical or clinical mastitis [7,27,28].

In conclusion, ion selective probes are sufficiently accurate to determine secretory activation by measurement of Na and K in the milk of lactating women. The use of ion selective probes may provide a useful point of care instrument to diagnose low milk supply and/or mammary infection. Early detection of these issues would allow timely intervention to ensure a successful lactation.

## Figures and Tables

**Table 1:** Participant Characteristics.

*n* = 65	Mean	Standard Deviation	Range
Maternal age (years)	34	4	24–43
Length of gestation (weeks)	39	2	30–41
Stage of lactation (weeks)	10	5	0.5–27
24 h milk production (millilitres)	780	318	36–1932

**Table 2:** Calculated Na:K ratio with or without the mean difference of K measurement between ISE and ICP-OES.

	Na (mM)	K (mM)	K + 0.65 mM	Na:K Ratio	Na:K + 0.65 mM Ratio
Low K	60	4	4.65	15.00	12.90
	40	4	4.65	10.00	8.60
	20	4	4.65	5.00	4.30
	12	4	4.65	3.00	2.58
Medium K	60	12	12.65	5.00	4.74
	40	12	12.65	3.33	3.16
	20	12	12.65	1.67	1.58
	12	12	12.65	1.00	0.95
High K	60	18	18.65	3.33	3.22
	40	18	18.65	2.22	2.14
	20	18	18.65	1.11	1.07
	12	18	18.65	0.67	0.64

**Table 3:** Human milk (HM) sodium and potassium concentrations measured using ion selective electrode (ISE) and inductively coupled plasma optical emission spectrometry (ICP-OES).

*n* = 65	Sodium (mM)			Potassium (mM)		
	ISE	ICP-OES	*p* value	ISE	ICP-OES	*p* value
Mean SD	6.18 2.47	5.91 3.37	0.20	11.70 2.21	11.10 1.55	0.01
Range	3.59–19.80	2.59–21.50		7.69–18.10	7.91–15.20	

**Table 4:** Limits of agreement for the measurement of Na and K concentrations using ICP-OES and the ion selective electrode.

Element	Mean Difference	CI mean Difference	Limits of Agreement		CI of Limits of Agreement	
			Lower	Upper	Lower	Upper
Na (mM)	0.26	−0.15, 0.68	−3.08	3.61	−3.81, −2.37	2.90, 4.34
K (mM)	0.65	0.17, 1.13	−3.19	4.49	−4.01, −2.37	3.67, 5.31

CI—95% confidence interval.
